# Unveiling residual, spontaneous recovery from subtle hemispatial neglect three years after stroke

**DOI:** 10.3389/fnhum.2015.00413

**Published:** 2015-07-30

**Authors:** Mario Bonato

**Affiliations:** Department of Experimental Psychology, Ghent UniversityGhent, Belgium

**Keywords:** stroke, hemispatial neglect, spontaneous recovery, spatial attention, spatial awareness, attentional load, neuroplasticity, attentional capacity

## Abstract

A common and disabling consequence of stroke is the difficulty in processing contralesional space (i.e., hemispatial neglect). According to paper-and-pencil tests, neglect remits or stabilizes in severity within a few months after a brain injury. This arbitrary temporal limit, however, is at odds with neglect’s well-known dependency on task-sensitivity. The present study tested the hypothesis that the putative early resolution of neglect might be due to the insensitivity of testing methods rather than to the lack of spontaneous recovery at later stages. A right hemisphere stroke patient was studied longitudinally for 3 years. According to paper-and-pencil tests the patient showed no symptom of hemispatial neglect 1 month post stroke. Awareness of spatially lateralized visual targets was then assessed by means of computer-based single- and dual-tasks requiring an additional top-down deployment of attention for the parallel processing of visual or auditory stimuli. Errorless performance at computer-based tasks was reached at month 12 and maintained until month 29 after stroke. A bottom-up manipulation was then implemented by reducing target diameter. Following this change, more than 50% of contralesional targets were omitted, mostly under dual-tasking. At months 40 and 41 the same task revealed a significant (but not complete) reduction in the number of contralesional omissions. Ipsilesional targets were, in contrast, still errorless detected. The coupling of a bottom-up (target change) and a top-down (dual-tasking) manipulation revealed the presence of a long-lasting spontaneous recovery from contralesional spatial awareness deficits. In contrast, neither manipulation was effective when implemented separately. After having excluded the potential confound of practice effects, it was concluded that not only the presence but also the time course of hemispatial neglect strongly depends on the degree of attentional engagement required by the task.

Right-hemisphere lesions often result in hemispatial neglect, a deficit affecting the conscious processing of the side of space opposite to the damaged hemisphere (Driver and Vuilleumier, 2001; Corbetta and Shulman, 2011; Bartolomeo et al., 2012; Vuilleumier, 2013). Neglect is a major burden for patients and families (Paolucci et al., 2001). Its rehabilitation poses a number of challenges to patients, clinicians and researchers (Barrett et al., 2006). In the case of clinicians and researchers these challenges are largely due to the heterogeneity of factors influencing this disorder (Bowen et al., 1999). Just to provide two examples, the presence and the severity of neglect dissociate depending on the nature of the task (e.g., cancellation vs. line bisection, see Ferber and Karnath, 2001) and spatial domain under investigation (e.g., peripersonal vs. extrapersonal space, Halligan and Marshall, 1991).

In addition to these well-documented factors, several lines of evidence have recently converged on the notion that the presence and the severity of neglect strikingly depends on the difficulty level of the task at hand. Specifically, the level of attentional engagement required by the task and the (im)possibility to actively compensate for the spatial attention deficits are major determinants of neglect (Bartolomeo, 1997; Rengachary et al., 2009; Bonato et al., 2010; Bonato and Deouell, 2013; van Kessel et al., 2013). Increased attentional engagement can hamper contralesional awareness in two different ways: by enlarging the degree of neglected contralesional space (Russell et al., 2004; Sarri et al., 2009) or, when the eccentricity of targets is kept constant, by increasing the proportion of omissions (Bonato et al., 2013). The dependence of contralesional hemispace deficits on task difficulty clearly depicts neglect as a continuous rather than dichotomous disorder. According to this view, some patients with non-pathological scores on tests might simply suffer mild neglect which goes undetected by standard methods. This issue is frequently overlooked when studying the evolution of neglect over time. Current knowledge about neglect’s spontaneous remission has been acquired by using the same paper-and-pencil tests which are currently considered insufficiently sensitive to detect its presence (Rengachary et al., 2009). Unsurprisingly, these tests show an initial, fast recovery (Nijboer et al., 2013) which is followed, within 2 to 4 months after insult, by either a stabilization or a disappearance of neglect (Cassidy et al., 1998). In the majority of patients, including those cases with severe spatial neglect in the acute phase, performance is thought to recover and stabilize by the third month post stroke onset (Stone et al., 1992). However, an even faster recovery rate has been reported. According to Robertson and Eglin (1992), “clinical neglect usually lasts for only a few weeks or months” (p. 169). As reported in Maguire and Ogden (2002), full resolution of symptoms can occur in most patients even earlier, i.e., over the first 10 days. In contrast to the studies above, other authors (Denes et al., 1982) suggest that neglect is much more stable over time and that only a minority of patients fully recover within 6 months. Nevertheless, both views agree on the absence of spontaneous recovery at later stages.

Here I aim to provide a proof of concept for the notion that the putative invariance of neglect symptoms in chronic stroke survivors reflects task insensitivity rather than genuine stabilization/remission of deficits. The present study reports on a right hemisphere stroke patient whose (apparent) recovery from visuospatial deficits during the post-acute phase, assessed with both standard tests and computerized tasks, has already been described in detail elsewhere (Bonato et al., 2012). In that study it was found that, despite a normal performance on paper-and-pencil tests, the requirement to perform a spatial monitoring task while concurrently responding to visual or auditory stimuli (i.e., dual-task setting) exposed the latent presence of neglect. The dual-task manipulation enabled the detection of a large number of contralesional omissions (>80%) one month after stroke, and the omissions rapidly decreased to below 25% by the third month. The crucial aspect of the study by Bonato et al. (2012) was that, although omissions could not be detected with clinical tests, they were nonetheless detected through computer-based dual-tasking. However, as reviewed above, detecting spontaneous recovery within the first few months after stroke is not surprising (see Kwakkel and Kollen, 2013, for a review).

The natural continuation of the study by Bonato et al. (2012) would be an investigation, in the same patient, of recovery at later stages, when spontaneous recovery is considered rare, and neuropsychological data on spatial processing improvements are hardly ever longitudinally collected (e.g., Karnath, 1988). Even considering research performed on earlier stages (e.g., few weeks to months post injury), to my knowledge, there is no study that took practice effects into account when the methods involved repeated administrations of paper-and-pencil tests such as drawing and cancellation. Thus, it is difficult to discern whether performance improvements are due to deficit remission or to test-retest practices. In the present study, I examined the hypothesis that (latent forms of) neglect can be revealed by increasing processing load. A stronger attentional engagement, in the case of latent spatial disorders, would then results in reduction of attentional capacity specific to the contralesional hemispace. The hypothesis was tested using a single vs. dual-task approach (Bonato et al., 2012). Computer-based tasks have the potential to unveil the presence of spatial neglect even several years post injury (Farnè et al., 2004; Deouell et al., 2005; Bonato, 2012).

## Method

The research was approved by the Ethical committee of the Department of General Psychology, University of Padua. The patient gave informed consent to take part in the study in accordance with the Declaration of Helsinki.

### Case Details

**Figure 1:**
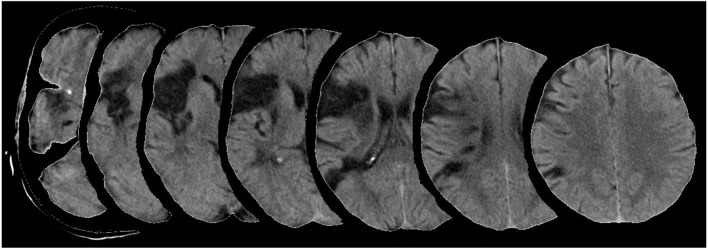
**CT scans of patient GB after 3 months from stroke onset.** Fronto-parietal hypointensity within the territory of the right middle cerebral artery is visible.

The patient (hereafter: GB) was a right-handed woman who had a stroke at the age of 63 years. Her brain scans collected 3 months after stroke (see Figure 1) showed a vast cortical-subcortical ischemic area which included the frontal pars opercolaris, the insula, and the posterior parietal cortex within the right middle cerebral artery territory. She underwent thrombolysis. One month after stroke her performance on a comprehensive battery for neglect (Behavioral Inattention Test, BIT, Wilson et al., 1987) was within normal range. Despite the considerable lesion, the patient had no residual motor disorders at discharge.

### Procedures

This paper reports on the results of six testing sessions which took place at patient’s home after 328 days (month 11), 476 days (month 16), 886 days (month 29), 1221/1222 days (month 40, session 1 and 2) and 1254 days (month 41) after stroke. In all sessions, each lasting approximately 2 h, GB was asked to perform three computer-based tasks and two Double-Stimulus Stimulation tasks.

Her performance between month 1 and month 6 after stroke was reported previously (Bonato et al., 2012).

### Computer-Based Single and Dual Tasks

Computer-based tasks were programmed with E-Prime.^1^ A head-and-chin rest was placed at a distance of 60 cm from a 15″ computer monitor. A camcorder recording enabled the detection of trials contaminated by eye movements. Each trial started with a blank screen (1000 ms) followed by a central, black fixation point (1000 ms, white background) and encompassed the concurrent, brief (50 ms) visual presentation of a lateralized target dot and of a letter at fixation (a, b, v, or z, see Bonato et al., 2012). Concurrently, a spoken number word (e.g., one, two, eight, or nine) was presented through headphones. The target could appear with the same probability on the left, on the right or bilaterally with a distance from the center of 13°. In all testing sessions performed until month 29 (included), the target diameter was 8 mm and subtended 0.8° of visual angle. From the second task administration at month 29 onwards, its diameter was then reduced to 3 mm (subtended angle about 0.3°).

**Figure 2:**
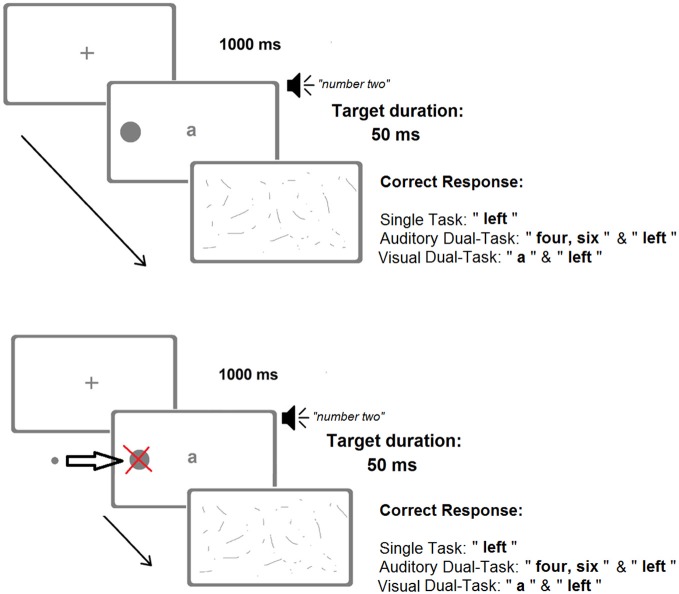
**A schematic representation of the three different conditions characterizing the main computer-based task.** The upper panel shows the large diameter (8 mm) version, administered until month 29. The lower panel shows the small diameter (3 mm) version, administered on month 29 and onwards. Adapted from Bonato (2012).

There were three conditions with identical stimuli and different instructions (Figure 2). Total duration was roughly 45 min. Task instructions and order were as follows:

In the single-task (ST) condition, the position of the target(s) (i.e., “right, “left, or “both” sides) had to be reported, disregarding the central letter and the auditory number.

In the auditory dual-task (ADT) condition, starting from the spoken number word (e.g., eight) the patient had to count forward twice in steps of two (e.g., ten-twelve), and then report the position of the lateral visual target(s).

In the visual dual-task (VDT) condition the spoken number word had to be ignored, and the centrally presented letter read aloud before reporting the position of the lateral visual target(s).

The experimenter coded the oral responses to the position of the target (“left”, “right”, “both” sides, no response) and to the concurrent task. Only trials with a correct response to the concurrent feature (if required) were considered. There were two blocks (48 trials each) in every experimental condition, for a total of 288 trials per session.

At month 40 a version encompassing additional no-target trials (25% of total) was presented to control for potential response bias. At month 41 the task was re-administered in the original, large diameter version. Two additional versions were also tested. In the first one the diameter of the left target was varied during the experiment, whereas in the second one the position of the left target was shifted either upwards or downwards (for 25% of vertical height).

Following Heilman et al.’s (2003, p. 296) definition neglect is “the failure to report, respond or orient to novel or meaningful stimuli presented to the side opposite a brain lesion, when this failure cannot be attributed to either sensory or motor deficits”. This definition will be operationalized by comparing omissions occurring in the contralesional hemispace across conditions as well as through the test of comparison with omissions occurring in the ipsilesional hemispace.

For each session the patient also underwent a Double-Stimulus Stimulation (DSS) in both the visual and the tactile modalities (Bisiach and Faglioni, 1974; see Maravita et al., 2007 regarding its high sensitivity). In the visual modality 20 bilateral, 32 left, and 32 right stimuli, half in the upper and half in the lower visual field, were presented in a fixed random order (Bisiach et al., 1983). In the tactile modality 10 bilateral, 16 left and 16 right stimuli were presented.

To ensure that healthy adults do not demonstrate lateralized bias in the current protocol, an age- and sex-matched healthy control was tested on the same task (small diameter version only).

## Results and Discussion

### Computer-Based Tasks

**Figure 3:**
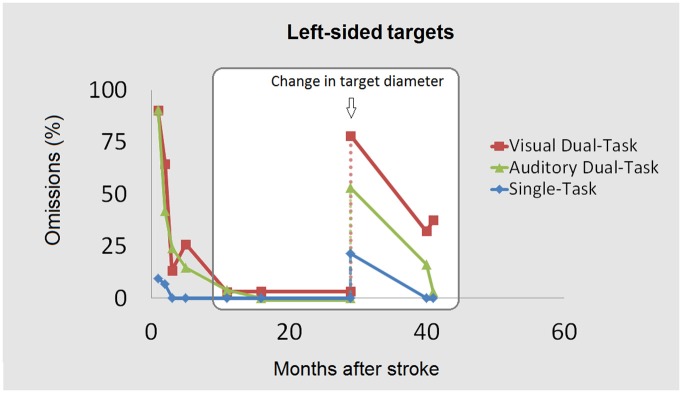
**The contralesional omissions (%) across the three main tasks (ST, ADT, VDT) are shown as a function of the temporal interval between the stroke and the testing.** The first three testing sessions (left) within the white square show performance following the presentation of a target with a diameter of 8 mm. The last three testing sessions show omissions obtained with the same tasks (single- or dual-) after target diameter reduction (3 mm). Additional variants (not reported here) show that the effects of response bias/test-retest effects were either limited or absent, confirming that omissions mainly indexed spontaneous recovery. Data points on the left of the square (recovery at earlier stages) are adapted from Bonato et al. (2012).

Trials contaminated by eye movements (<2%) were discarded from further analysis. Within each task trials were collapsed according to stimulus position. Analysis were performed for unilateral targets only. During the testing sessions performed at months 11, 16 and 29 using standard target diameter, GB showed virtually no contralesional omissions under any condition (average 1.5%, all *p*s *ns*, see Figure 3). This absence of omissions is the natural continuation of the decrease in the number of omissions which had been measured by Bonato et al. (2012) with the same tasks in the acute and post-acute phases (until month 6). However, it is also possible that this absence of omissions simply indicates that the task was no longer sufficiently engaging attention to reveal significant spatial asymmetries in attentional capacity. Accordingly, spontaneous yet more subtle recovery might still have been present.

#### Reduced Target Diameter

**Figure 4:**
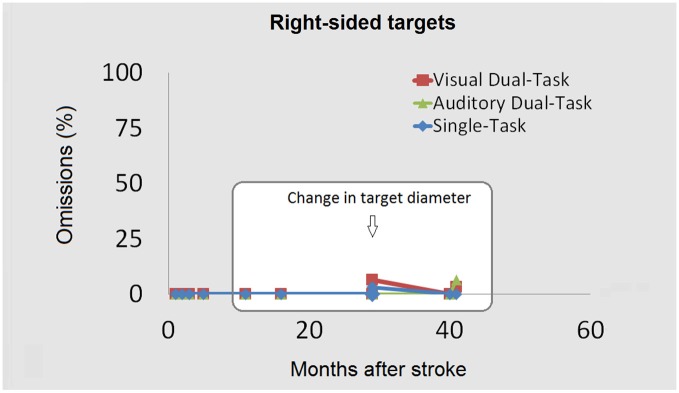
**The ipsilesional omissions (%) across the three main tasks (ST, ADT, VDT) are shown as a function of the temporal interval between the stroke and the testing.** The first three testing sessions (left) within the white square show performance obtained by presenting an 8 mm target. The last three testing sessions show omissions obtained with the same tasks (single- or dual-) after target diameter reduction.

In order to test whether the recovery measured by the task was full or only partial, target diameter was reduced (from 0.8° to 0.3°) and the task re-administered (months 29, 40 and 41). After the reduction in target diameter, at month 29, contralesional omissions re-emerged (see Figure 3). They were significantly more frequent in the VDT (78.1%) and in the ADT (53.1%) with respect to the ST (21.4%), *χ*^2^_(1,60)_ = 19.22, *p* < 0.001 and *χ*^2^_(1,57)_ = 8.19, *p* < 0.01, respectively. In other words, a rather striking number of omissions emerged across the very same tasks which, in the same session, led to ceiling performance when a larger target diameter was adopted. Significantly more omissions emerged for left than for right targets in the ST (3.1% of omissions) *χ*^2^_(1,59)_ = 4.6, *p* < 0.05; in the VDT (6.3%) *χ*^2^_(1,64)_ = 33.9, *p* < 0.001; and in the ADT (0%) *χ*^2^_(1,60)_ = 25.4, *p* < 0.001 (see Figure 4). By month 40 the number of contralesional omissions significantly decreased to 0% in the ST, *χ*^2^_(1,60)_ = 7.6, *p* < 0.05, to 32.3% in the VDT, *χ*^2^_(1,61)_ = 11.8, *p* < 0.001 and to 16.1% in the ADT, *χ*^2^_(1,49)_ = 5.4, *p* < 0.05. The difference with respect to right targets was significant for the VDT only (ipsilesional omissions = 0%), *χ*^2^_(1,60)_ = 12.8, *p* < 0.001 (see Figure 3). To exclude that these improvements were due to practice effects or to a response bias a version encompassing a condition without targets (25% of trials) was administered the following day, that is, during testing session 2 of month 40. Contralesional detection was still largely impaired under Dual-Task conditions (53.1 and 19.2% of omissions in the VDT and in the ADT, respectively, not shown in the Figure 3). Performance did not differ from session 1 (both *p*s > 0.2, *ns*). Moreover, no response bias emerged; across all trials without target, the patient refrained from responding (accuracy 100%).

After one month (41 from stroke), the reduced diameter task was re-administered. During this final session (see Figure 3) contralesional omissions found under VDT (37.5%) did not differ, *χ*^2^_(1,61)_ = 0.06, *p* = 1, *ns* compared with the month 40 session. There were no omissions under ST and a negligible number (3.1%, *ns*) under ADT. Only in the VDT condition were there significantly more omissions in the left than in the right (3.1%) hemispace, *χ*^2^_(1,63)_ = 11.3, *p* < 0.01 (see Figure 4).

#### Additional Tasks at Month 41

The version with larger target diameter was re-administered and again led to ceiling performance (0% omissions).

Two additional manipulations were then performed (not shown in the Figures 3, 4). The first one was aimed at further testing the reliability of the apparent causal link between reduced target diameter/dual-tasking and increased omissions. A modified VDT version was administered, encompassing the larger diameter for half of the left targets and the reduced diameter for the other half. A larger number of omissions were found for the small (62.5%) than for the full-sized (13.3%) targets, *χ*^2^_(1,34)_ = 9.8, *p* < 0.01.

The second manipulation was meant to assess the potential impact of practice effects in a different manner. The position of the left target was changed along the Y axis, with the target presented either in the upper or in the lower quadrant. If performance improvements were due to repetitive focusing of spatial attention at the only contralesional location where the target was expected (at this point of the testing GB had performed more than 3500 trials), adding two potential locations would have resulted in substantial performance deterioration.

However, the mean error rate resulting from this manipulation (33 or 37.1% considering DT conditions only) did not significantly differ from the 37.5% found for VDT, *χ*^2^_(1,63)_ = 0.87, *p* = 1, ns.

#### Excluding Within-Session Improvements

To directly exclude the presence of within-session improvements the data from the three testing sessions with reduced target diameter (months 29, 40 and 41) were re-analyzed and within session comparisons were performed. The number of omissions occurring within the first vs. the second block of every task was compared separately for each session. If anything, a tendency towards a fatigue effect was present (omissions first half: 21%; second half: 33%).

#### Control Participant

The control participant performed at ceiling across all tasks (100% of left targets detected). In a previous study (Bonato et al., 2010) the large diameter version has been administered to three healthy controls without resulting in any lateralized pattern of omissions.

#### Concurrent Task Performance

Concurrent task performance was at ceiling in the ADT and resulted in an accuracy as high as 99% in the VDT.

#### Double-Stimulus Stimulation

The patient correctly reported all contralesional single targets in both visual and tactile modalities across all sessions.

## General Discussion

The present study longitudinally tested the dependency of the spontaneous remission from neglect on task sensitivity in a right-hemisphere stroke patient. According to paper-and-pencil tasks, full recovery was already present 1 month after onset. According to performance on a computer-based task encompassing a top-down only manipulation, however, recovery completed only 12 months after stroke. It was here shown that, by adding to the same task a bottom-up manipulation leading to higher attentional engagement, enduring deficits were again detected during chronic phases (between month 29 and 41). Moreover, significant improvements still emerged across the various testing sessions.

The coupling of a top-down with a bottom-up manipulation was particularly effective. The number of omissions elicited at month 29 (small target) were similar to the number of omissions elicited at month 1 with the standard target. The presence of contralesional omissions was rather surprising for several reasons. First, they occurred at a very late stage (between 2.5 and 3.5 years from onset). Second, omissions emerged following repeated ceiling performance with the same task, which already successfully detected a “first”, seemingly full, remission in the post-acute phase. Third, the patient had no impairments according to clinical tests (paper-and-pencil tasks and DSS). Moreover, practice effects were excluded and performance for targets appearing within the ipsilesional hemispace was at ceiling.

The detection of significant, spontaneous recovery after more than 3 years from lesion, when no spontaneous improvements can typically be measured, suggests that previous inferences about the short time course of neglect remission were biased by the use of rather insensitive instruments. The number of task variants allowed for the exclusion of alternative explanations and to conclude that the performance improvements mostly mirrored the remission of the core deficit suffered rather than compensatory strategies or test-retest practice effects as is often the case with classic paper-and-pencil tests.

In the current study, the bottom-up and top-down manipulations were implemented both concurrently and in isolation. One might have expected that after such a long post-injury temporal interval, either the dual task or the target-related manipulation would become ineffective. Yet, the data showed that both manipulations were ineffective when implemented in isolation but still led to a substantial number of omissions when concurrently implemented. In the cases both of dual-tasking with a larger target dimension and of single task with a small target dimension, omissions were almost or totally absent. The observation that omissions only emerged in a dual-task setting, confirms that high task engagement results in enhanced sensitivity in detecting spatial awareness biases. This sensitivity allowed to effectively induce and measure contralesional omissions even at very late stages post stroke. Even in healthy participants visuospatial processing is characterized by capacity limits (Morgan and Solomon, 2006; Holcombe and Chen, 2012; Giesbrecht et al., 2014). Dual-task taxing on attentional control highlights that the capacity limits become, after brain damage, spatially asymmetric with impaired performance in the contralesional hemispace (Habekost and Rostrup, 2006).

The similar degree of omissions across two dual-tasks which were very different in nature can also be easily interpreted by positing the presence of supramodal attentional resources whose amount is limited and depletable (see Bonato, 2012, for a review). The overlapping performance found for the two dual tasks in the acute stage diverged at the last stage (month 41), with worse performance for the VDT (stable omissions) than for the ADT (disappearance of omissions). This difference between dual-tasks indicates that, at least after repeated practice, VDT performance is more stable and immune from test-retests effects with respect to ADT. In general, the deterioration found under dual-tasking, in particular for the ADT, was neither obvious nor trivial. The opposite results might have been predicted because right-hemisphere patients are known to strikingly improve their spatial monitoring performance when alerted by a sound (Robertson et al., 1998). Moreover, when motor output is measured in neurological patients (or in older healthy adults, see Verrel et al., 2009), a cognitive dual-tasking can lead to improvements in performance due to the increased need to focus on the task and possibly also because of its more controlled implementation (Bourlon et al., 2014). The strong asymmetry between the consistent effects on the spatial monitoring and the absence of effect on the depleting task (Schaefer, 2014) allow us to identify the spatial monitoring as the cognitive ability mostly affected by the stroke. However, the unidirectional modulation of non-spatial tasks upon spatial awareness does not exclude that bidirectional influences are possible and that lateralized targets might modulate performance in non-spatial tasks. Indeed spatial and non-spatial deficits are closely intertwined in neglect (Van Vleet and DeGutis, 2013).

After a brain injury, in the absence of additional intervening factors, performance tends to improve with time, due to cerebral reorganization and the implementation of strategies. It is however rather difficult to distinguish these two closely associated components (Murphy and Corbett, 2009). The promising insensitivity of the instrument to test-retest effects described here might allow researchers to better focus on components that are due to genuine recovery rather than to practice effects and compensatory strategies. The increase in contralesional omissions when a concurrent target was ipsilesionally presented provides additional evidence of the pathological and asymmetric dependency of contralesional attentional capacity on attentional engagement and processing load induced by unilateral brain damage.

### Study Limitations

While the dual-tasking approach is promoted as a more appropriate testing option for spatial disorders in the chronic phase, its limitations should be clearly outlined and heeded.

First, it measures performance in the peripersonal spatial domain only, and does not provide any hints on performance in the personal nor in the extrapersonal domain. Second, the contralesional deficits detected by the dual-task/small target manipulation may not have a counterpart in everyday life. Further research is therefore needed to explore to what extent task performance correlates with everyday life performance. Third, a larger control group should be tested to further ensure that the deficits here described are caused by the brain damage and not by an asymmetry in attention (Peers et al., 2006).

### Clinical Implications

The findings from the present study provided empirical evidence for the hypothesis (Kwakkel et al., 2006) that the early disappearance of post-stroke improvements might be due to ceiling effects. According to performance on cancellation tasks, which are commonly adopted for neglect diagnosis (Ferber and Karnath, 2001), spontaneous neglect recovery ends approximately 3 months after stroke (Nijboer et al., 2013). In contrast, the attention-demanding approach adopted in the present study has demonstrated that improvement of neglect continues for more than 3 years post injury.

It is worth remembering that undiagnosed neglect precludes proper access to rehabilitation and constitutes a major threat for patients and for other people (Deouell et al., 2005). In the present study, the performance of GB was notably affected by neglect in everyday-life, although mostly evident only when she had to deal with dual-tasking situations in spatial contexts. At month 41, after the testing ended and while talking with the experimenter, GB failed to notice the presence of a glass with a beverage which was being offered by her husband on her left-hand side. The husband repeatedly invited her to grasp the glass but she failed to notice its presence. Notably, in the presence of motor or cognitive deficits (absent in GB but often present in stroke), it would have been very difficult to demonstrate any (subtle) neglect presence. Both GB and her husband denied any problem related to “forgetting the left side of space” in everyday life.

While taking into account the above mentioned limitations, it should however be noted that the VDT version conceptually resembles the peripheral target detection of the Useful Field Of View (UFOV; Ball et al., 1988) test and that, in turn, performance on the UFOV is an excellent predictor of car crash risk in the elderly (Owsley et al., 1998). Importantly, the current problem for neglect diagnosis concerns false negatives and not false positives. Indeed, despite its early disappearance from most clinical measures (Corbetta and Shulman, 2011), neglect is known to negatively influence the possibility for patients to return to a productive life (Denes et al., 1982; Paolucci et al., 2001).

The high sensitivity and specificity characterizing this new approach are crucial aspects for a neuropsychological instrument. Far from merely being an elegant manipulation, the impossibility of efficiently performing multitasking can have a negative impact on patients’ autonomy. Paradoxically, the dual-task, computer-based manipulation can be seen as closer to everyday life than paper-and-pencil tests (see Deouell et al., 2005 vs. Jehkonen et al., 2012). The idea here was not to use materials/settings one is familiar with, but rather, to simulate the cognitive demands everyday life requires. Surprisingly enough, the dual-task approach is widely adopted in clinical fields where cognitive components play a less prominent role. For instance, in the motor domain, the great sensitivity of dual-tasking approach for estimating the risk of falls (Schaefer, 2014) and walking performance after stroke (Baetens et al., 2013) is well-established. The difficult question of whether an impaired performance for the contralesional hemispace results in functional impairments can be answered only in a patient-specific manner. If a patient wants to return to driving, a subtle deficit like the one described here might potentially result in a functional impairment.

### Conclusion

The present research findings do not question but rather confirm that recovery is quantitatively larger and qualitatively more genuine in the early phases after stroke (Stone et al., 1992; Nijboer et al., 2013). What can be considered new and unequivocal is that different tasks leading to heterogeneous levels of attentional engagement, not necessarily spatial in nature, will result in very different mappings of recovery in time. The unexpectedly long-lasting (neuro)functional plasticity (Murphy and Corbett, 2009) revealed by this new approach also suggests that restitutive neurorehabilitation might be possible for several years after stroke. Moreover, highly sensitive approaches as the one described here can detect small post-rehabilitation improvements which would instead go undetected by standard, less sensitive, instruments.

## Conflict of Interest Statement

The author declares that the research was conducted in the absence of any commercial or financial relationships that could be construed as a potential conflict of interest.

## Footnotes

^1^http://www.pstnet.com/
